# Nicotianamine synthase overexpression positively modulates iron homeostasis-related genes in high iron rice

**DOI:** 10.3389/fpls.2013.00156

**Published:** 2013-05-29

**Authors:** Meng Wang, Wilhelm Gruissem, Navreet K. Bhullar

**Affiliations:** Plant Biotechnology, Department of Biology, Swiss Federal Institute of TechnologyZürich, Switzerland

**Keywords:** iron, homeostasis, NFP rice, biofortification, expression profiling

## Abstract

Nearly one-third of the world population, mostly women and children, suffer from iron malnutrition and its consequences, such as anemia or impaired mental development. Biofortification of rice, which is a staple crop for nearly half of the world's population, can significantly contribute in alleviating iron deficiency. NFP rice (transgenic rice expressing nicotianamine synthase, ferritin and phytase genes) has a more than six-fold increase in iron content in polished rice grains, resulting from the synergistic action of nicotianamine synthase (*NAS*) and ferritin transgenes. We investigated iron homeostasis in NFP plants by analyzing the expression of 28 endogenous rice genes known to be involved in the homeostasis of iron and other metals, in iron-deficient and iron-sufficient conditions. RNA was collected from different tissues (roots, flag leaves, grains) and at three developmental stages during grain filling. NFP plants showed increased sensitivity to iron-deficiency conditions and changes in the expression of endogenous genes involved in nicotianamine (NA) metabolism, in comparison to their non-transgenic siblings (NTS). Elevated transcript levels were detected in NFP plants for several iron transporters. In contrast, expression of *OsYSL2*, which encodes a member of yellow stripe like protein family, and a transporter of the NA-Fe(II) complex was reduced in NFP plants under low iron conditions, indicating that expression of *OsYSL2* is regulated by the endogenous iron status. Expression of the transgenes did not significantly affect overall iron homeostasis in NFP plants, which establishes the engineered push-pull mechanism as a suitable strategy to increase rice endosperm iron content.

## Introduction

Iron deficiency anemia (IDA) is the most severe degree of iron deficiency and a global problem that affects an estimated one-third of the world's population in both developing and developed countries. IDA has major consequences for human health as well as social and economic progress (WHO, 2013). Human IDA could be relieved by iron supplementation or food fortification. However, iron supplementation is difficult to achieve due to transportation and economic circumstances, especially in rural areas of developing countries. Iron fortification of food is also technically difficult and often results in unacceptable color and flavor of fortified products (Hurrell and Egli, 2010). In the recent years, bio-fortification has emerged as a possible solution to combat iron deficiency anemia through an economical and natural way.

Rice is the second largest produced cereal in the world and the most important grain with regard to human nutrition and caloric intake. It provides more than one fifth of the calories consumed worldwide. Around 3 billion people, mostly in Asia, depend on rice for 35–59% of their caloric intake. However, rice is a poor source of micronutrients, including iron. Most commercial rice varieties have only around 2 μg/g iron in the endosperm. Therefore, rice cannot provide daily iron needs of humans i.e., at least 8 mg/day for males and 18 mg/day for females, with pregnant women requirements rising up to 27 mg/day (Institute of Medicine, 2013). Considering these facts, enrichment of rice endosperm with bioavailable iron has the potential to decrease iron malnutrition worldwide. However, iron biofortification of rice strongly relies on information on the genes that control iron homeostasis in plants.

Iron translocation and homeostasis in rice has been well-studied. Several genes, most of which are transcriptionally regulated in response to iron availability, are known to coordinate iron uptake, translocation and storage in various tissues/compartments of the plant (Kobayashi and Nishizawa, 2012). However, the contribution of each type of transporter(s) and the precise iron flux still need to be clarified for each step involved in iron translocation. The transcription factors *OsIDEF1* and *OsIDEF2* regulate iron homeostasis-related genes in rice during Fe deficiency (Ogo et al., 2008; Kobayashi et al., 2009, 2010a). It has been suggested that *OsIDEF1* senses the cellular iron status by binding directly to the metal ions (Kobayashi et al., 2012). To cope with Fe starvation, rice roots release phytosiderophores (PS), which are molecules of the mugineic acid (MAs) family that form strong hexadentate chelates with Fe(III) to solubilize and transport it to the plant (Walker and Connolly, 2008; Palmer and Guerinot, 2009). The resulting Fe(III)-PS complexes are transported into root cells via transporters of the Yellow stripe like (YSL) family of proteins (Inoue et al., 2009; Lee et al., 2009a). Nicotianamine (NA), which is synthesized by nicotianamine synthase (NAS) from S-adenosyl-L-methionine, is a ubiquitous metal chelator in plants and regulates iron translocation within and between cells and transports it to veins, flowers, and seeds (Takahashi et al., 2003). NA also serves as a substrate for nicotianamine aminotransferase (NAAT) to produce a 3″-oxo intermediate and subsequently, DMA is synthesized by deoxymugineic acid synthase (DMAS) (Haydon and Cobbett, 2007; Kim and Guerinot, 2007). Six members of *OsNAAT* family have been identified in rice plants, however, only *OsNAAT1* is regulated by plant iron status (Inoue et al., 2008). *OsDMAS1* is also up-regulated in both root and shoots under iron-deficient condition (Bashir et al., 2006; Bashir and Nishizawa, 2006). In addition to the iron uptake using phytosiderophores/DMA, rice also possesses an Fe(II) uptake system. *OsIRT1* and *OsIRT2*, the homologs of iron-regulated transporter *IRT1* in Arabidopsis, are specifically up-regulated in roots of iron-deficient rice plants (Ishimaru et al., 2006).

Once iron is loaded into the xylem, the chelators such as citrate, NA, and DMA are required for further transport in the plant (Jeong and Guerinot, 2009). In rice, a ferric reductase defective (*FRD*) *3*-like gene, *OsFRDL1*, is involved in iron-citrate translocation from rice roots to shoots (Yokosho et al., 2009). Transporters that are encoded by the YSL family of genes, *OsYSL2*, *OsYSL15*, *OsYSL16*, *OsYSL18*, are also involved in long distance transport of DMA-Fe(III) and/or NA-Fe(II) complexes (Aoyama et al., 2009; Inoue et al., 2009; Ishimaru et al., 2010; Kakei et al., 2012; Zheng et al., 2012). However, much still remains to be unraveled about intracellular metal transport involving vacuoles, chloroplasts, and mitochondria, although some transporters have been identified with specific iron translocation roles for these compartments. An iron deficiency-inducible mitochondrial iron-regulated gene (*OsMIR*; Ishimaru et al., 2009) and mitochondrial iron transporter (*OsMIT*), whose expression increases under excessive iron condition, were identified in rice (Bashir et al., 2011). Permease in chloroplasts 1 (*OsPIC1*) is also associated with chloroplast iron transport, while the vacuolar membrane localized *OsVIT1* and *OsVIT2* (Vacuolar iron transporter 1 and 2) mediate sequestration of Fe(II), Zn(II), and Mn(II) into vacuoles, with *OsVIT2* being very responsive to Fe treatments (Zhang et al., 2012). Conversely, transporters of the Natural Resistance Associated Macrophage Protein (NRAMP) family appear to have important roles in mobilizing export of vacuolar Fe stores (Lanquar et al., 2005). Despite these advancements, the coordinated function of different transporters that have a role in iron homeostasis is not fully understood.

Strategies to improve iron content in rice grains were mostly targeted at effective iron (Fe) uptake from the soil and translocation in the plant, in addition to directing Fe into the rice endosperm. Most of the strategies used NAS and ferritin, a protein that stores iron in a bioavailable form (Lonnerdal et al., 2006; Jin et al., 2009). Endosperm-specific expression of ferritin or the constitutive expression of NAS mostly achieved around 2- to 3-fold increases of iron in the endosperm (Goto et al., 1999; Lucca et al., 2001; Vasconcelos et al., 2003; Qu et al., 2005; Lee et al., 2009b, 2012). A 4.2-fold increase in Fe content was reported in plants over-expressing *OsNAS2* under the control of the CaMV35S promoter (Johnson et al., 2011). The possibility of using other transporters for improving endosperm iron content has also been explored recently. Specific expression of *OsYSL2* in the vascular tissue and around the endosperm lead to a 4.4-fold increase of iron concentration in the polished rice grains (Ishimaru et al., 2010). Over-expression of *OsIRT1* under the control of the maize ubiquitin promoter also increased Fe concentration to 113% compared to wild type grains (Lee and An, 2009). Alternatively, a few studies focused on the endosperm-specific expression of *Phytases* (Lucca et al., 2001). These enzymes can degrade phytate, a chelating agent that binds iron as well as other metals and store them in a non-bioavailable form for human consumption within the grain (Brinch-Pedersen et al., 2002).

The overexpression of multiple genes through a single construct, i.e., barley NAS expressed under rice actin promoter, soybean ferritin duplicated and expressed under two different endosperm specific promoters as well as rice *OsYSL2* duplicated and expressed under endosperm specific and sucrose transporter promoters, resulted in 4.4-fold increase of iron in polished grains of field grown T3 rice plants (Masuda et al., 2012). In another approach, Wirth and collaborators reported a more than 6-fold increase in endosperm of rice plants constitutively expressing *A. thaliana NAS* (*AtNAS*), together with endosperm-specific expression of *Phaseolus vulgaris* ferritin and *Aspergillus fumigates* phytase as a single construct (NFP rice; Wirth et al., 2009). The effect of NAS and ferritin genes was synergistic in these plants, indicating that none of the iron uptake, transport, or storage systems in the engineered rice plants were saturated.

Here, we investigated the molecular impact of the transgenes on the expression of endogenous iron homeostasis-related genes in the engineered NFP rice plants. We performed targeted expression profiling of 28 genes involved in iron homeostasis. Our data suggests that the transgenes did not interfere with endogenous iron homeostasis at large, but modulated the expression of a few genes to facilitate iron uptake, translocation, and storage. The results provide new insights into coordinated role of different genes, particularly those involved in phytosiderophore synthesis and iron translocation, in maintaining iron homeostasis within the NFP plants while transporting more iron to the grains in these plants.

## Results

The relative expression levels of 28 endogenous rice genes related to iron (or metal) homeostasis (Table 1) were analyzed in transgenic NFP plants and non-transgenic control plants (NTS). The genes studied included those involved in NA and DMA synthesis, the YSL transporters, the iron-regulated transporters, genes from zinc-regulated transporter IRT-like proteins (ZIP) family, transcription factors, as well as the inter- and intra-cellular transporters. The plants were subjected to sufficient and deficient iron availability conditions and the expression levels of selected genes were studied in flag leaf, root, and grain samples collected at three grain development stages, i.e., milky, dough, and mature. In order to select for the reference genes which could be used for all the different sample types and growth stages, a preliminary test with at least 13 genes selected from the gene expression database, Genevestigator™ (Zimmermann et al., 2005) as well as literature were tested. The genes with medium to high expression in all rice tissues were chosen from Genevestigator™ for the pilot qRT-PCR test. Among these tested genes, IWS1 C-terminus family protein (LOC_Os01g05420) and ATP binding protein (LOC_Os11g43970.1) ranked among the best five genes identified in our analysis and were therefore used in the experiment. The data from LOC_Os01g05420 expression was used for normalization of real-time quantitative expression of the test genes. The observed changes in the expression patterns of the tested genes in the NFP plants in comparison to their non-transgenic siblings (NTS plants) are summarized below.

**Table 1:** **List of genes tested for their expression pattern in the NFP plants in comparison to their non-transgenic siblings**.

**Genes tested**	**Root**	**Leaf**	**Grain**	**References**
**PHYTOSIDEROPHORES SYNTHESIS RELATED GENES**				
S-adenosyl methionine synthetase 2 (*OsSAMS2*)	X	X	X	Lee et al., 1997
Nicotianamine synthase 1 (*OsNAS1*)	X	X		Inoue et al., 2003; Kobayashi et al., 2005
Nicotianamine synthase 2 (*OsNAS2*)	X	X	X	Inoue et al., 2003; Kobayashi et al., 2005
Nicotianamine synthase 3 (*OsNAS3*)	X	X		Inoue et al., 2003
Nicotianamine aminotransferase (*OsNAAT1*)	X	X	X	Inoue et al., 2008
Deoxymugineic acid synthase (*OsDMAS1*)	X	X	X	Bashir et al., 2006
**INTER- AND INTRA-CELLULAR METAL TRANSPORTERS AND OTHER IRON RESPONSIVE GENES**				
Iron-regulated transporter 1 (*OsIRT1*)	X	X	X	Ishimaru et al., 2006
Iron-regulated transporter 2 (*OsIRT2*)	X	X		Ishimaru et al., 2006
Yellow stripe like 2 (*OsYSL2*)	X	X	X	Koike et al., 2004
Yellow stripe like 5 (*OsYSL5*)	X	X		Narayanan et al., 2007
Yellow stripe like 6 (*OsYSL6*)	X	X	X	Narayanan et al., 2007
Yellow stripe like 9 (*OsYSL9*)	X	X		Aoyama et al., 2009
Yellow stripe like 13 (*OsYSL13*)	X	X		Nozoye et al., 2011
Yellow stripe like 15 (*OsYSL15*)	X			Inoue et al., 2009
Zinc-regulated transporter, Iron-regulated transporter-like 1 (*OsZIP1*)	X	X		Ramesh et al., 2003
Zinc-regulated transporter, Iron-regulated transporter-like 3 (*OsZIP3*)	X	X		Ramesh et al., 2003
Zinc-regulated transporter, Iron-regulated transporter-like 4 (*OsZIP4*)	X	X	X	Ishimaru et al., 2005
Zinc-regulated transporter, Iron-regulated transporter-like 8 (*OsZIP8*)	X			Narayanan et al., 2007
Heavy metal ATPase 2 (*OsHMA2*)	X	X		Takahashi et al., 2012
Ferric reductase defective 3 like (*OsFRDL1*)	X			Yokosho et al., 2009
Natural resistance associated macrophage protein 4 (*OsNRAMP4*)	X	X		Narayanan et al., 2007
Mitochondrial iron-regulated gene (*OsMIR*)	X	X	X	Ishimaru et al., 2009
Metal tolerance protein 1 (*OsMTP1*)	X	X		Yuan et al., 2012
Vacuolar iron transporter 2 (*OsVIT2*)	X	X	X	Zhang et al., 2012
Permease in chloroplasts1 (*OsPIC1*)		X	X	Duy et al., 2007
**IRON STORAGE PROTEIN FERRITIN AND TRANSCRIPTION FACTORS**				
Ferritin 1 (*OsFER1*)	X	X	X	Stein et al., 2009
*IDEF1*	X	X	X	Kobayashi et al., 2009, 2012
*IDEF2*	X	X	X	Ogo et al., 2008

The symbol “X” marks the tissue (root, flag leaf, or grain) in which the corresponding gene was tested.

### Expression profiles of genes involved in phytosiderophore synthesis and iron uptake in roots

**Figure 1:**
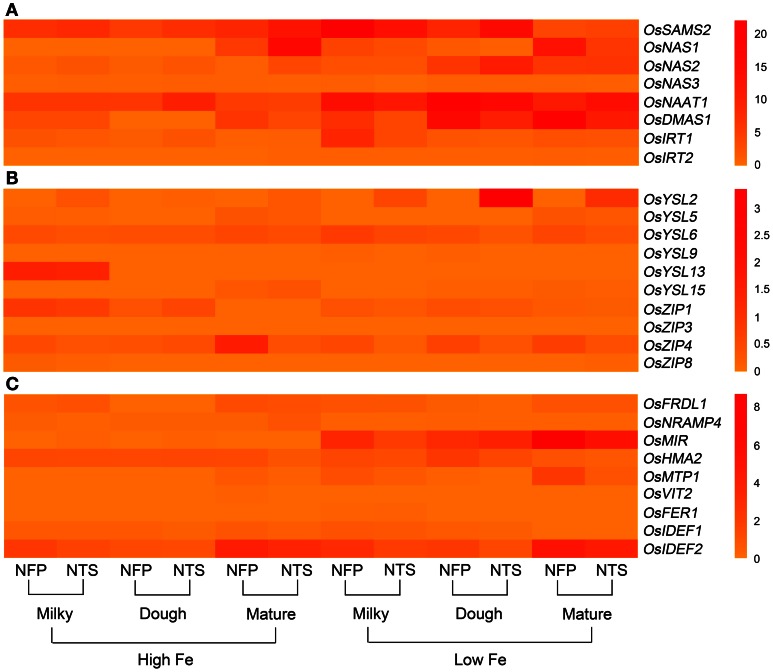
**Overall view of genes that are differentially expressed in NFP roots as compared to NTS roots over different plant development stages (corresponding to grain filling stages—milky, dough, and mature) and upon different iron supplies (high and low Fe). (A)** Expression levels of genes involved in nicotianamine and deoxymugeneic acid synthesis as well as the iron-regulated transporters. **(B)** Expression levels of genes belonging to yellow stripe like family of transporters and the transporters belonging to zinc-regulated transporter IRT-like proteins (ZIP) family. **(C)** Expression levels of transcription factors *OsIDEF1* and *OsIDEF2* regulating iron homeostasis as well as other inter- and intra-cellular metal transporters. Data represents the mean of three biological replicates. Note the differences in scale between panels **(A), (B),** and **(C)** for relative expression levels of tested genes.

**Figure 2:**
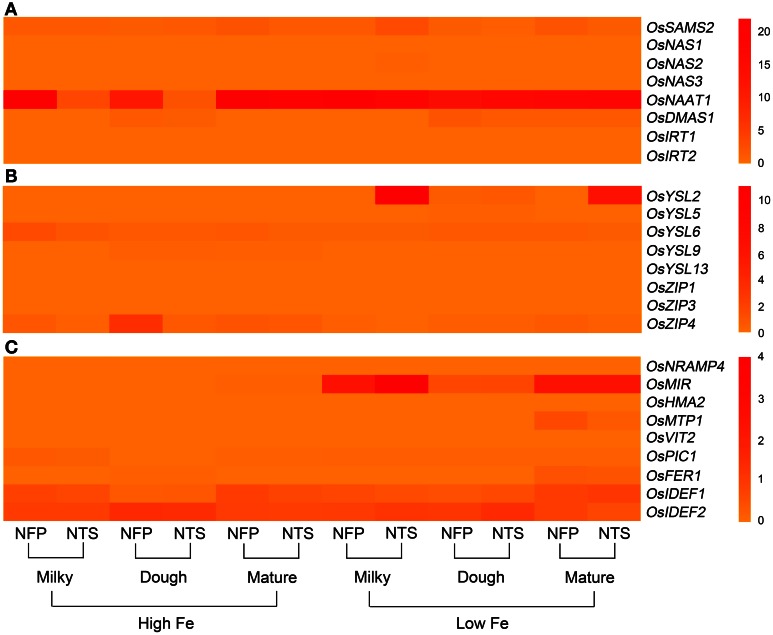
**Overall view of genes that are differentially expressed in NFP flag leaves as compared to NTS flag leaves over different plant development stages (corresponding to grain filling stages—milky, dough, and mature) and upon different iron supplies (high and low Fe). (A)** Expression levels of genes involved in nicotianamine and deoxymugeneic acid synthesis as well as the iron-regulated transporters. **(B)** Expression levels of genes belonging to yellow stripe like family of transporters and the transporters belonging to zinc-regulated transporter IRT-like proteins (ZIP) family. **(C)** Expression levels of transcription factors *OsIDEF1* and *OsIDEF2* regulating iron homeostasis as well as other inter- and intra-cellular metal transporters. Data represents the mean of three biological replicates. Note the differences in scale between panels **(A), (B),** and **(C)** for relative expression levels of tested genes.

**Figure 3:**
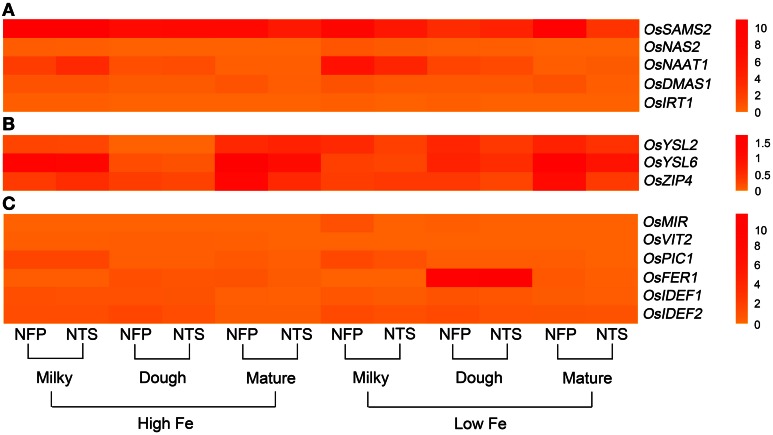
**Overall view of genes that are differentially expressed in NFP grains as compared to NTS grains over different plant development stages (corresponding to grain filling stages—milky, dough, and mature) and upon different iron supplies (high and low Fe). (A)** Expression levels of genes involved in nicotianamine and deoxymugeneic acid synthesis as well as the iron-regulated transporters. **(B)** Expression levels of genes belonging to yellow stripe like family of transporters and the transporters belonging to zinc-regulated transporter IRT-like proteins (ZIP) family. **(C)** Expression levels of transcription factors *OsIDEF1* and *OsIDEF2* as well as other inter- and intra-cellular metal transporters. Data represents the mean of three biological replicates. Note the differences in scale between panels **(A), (B),** and **(C)** for relative expression levels of tested genes.

**Figure A1:**
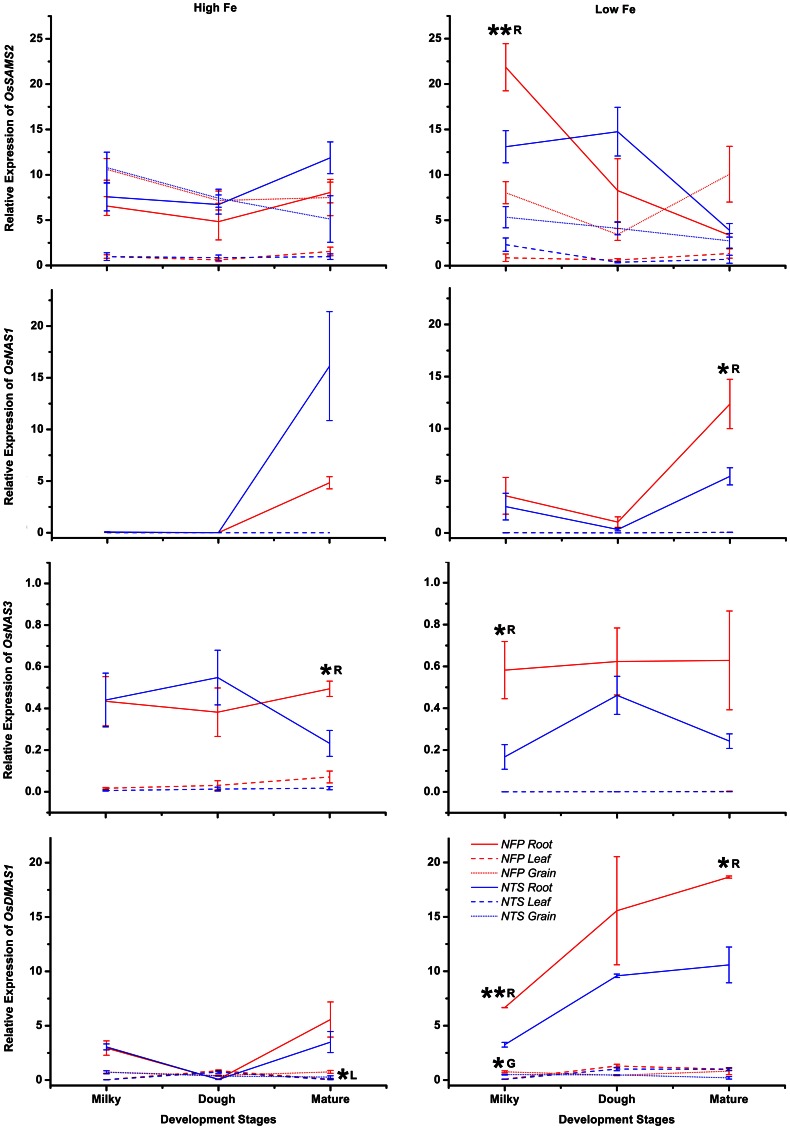
**Differences in expression levels for genes involved in nicotianamine and deoxymugeneic acid synthesis (*OsSAMS2*, *OsNAS1*, *OsNAS3*, and *OsDMAS1*), in the roots, flag leaves, and grains of NFP vs. NTS plants are presented.** Values are mean ± standard error of mean, of the three biological replicates. Significant differences between NFP and NTS tissues (roots, flag leaves, grains) under a particular treatment (high Fe or low Fe) or at a particular development stage (milky, dough, or mature) are marked with single (^*^p ≤ 0.05) or double asterisks (^**^p ≤ 0.01), respectively. The letters R, L, and G represent root, leaf, and grain, respectively. Note the differences in scale at Y-axis for relative expression levels of tested genes.

Six rice genes encoding enzymes in phytosiderophore synthesis, and thus involved in iron uptake as well as in iron translocation, were studied. The genes included S-adenosyl-l-methionine synthetase 2 (*OsSAMS2*), the NAS family members *OsNAS1*, *OsNAS2* and *OsNAS3*, *OsNAAT1*, as well as *OsDMAS1*. The genes (except *OsNAS3*) are predominantly expressed in roots and induced by low Fe availability as a part of the Fe deficiency response that serves to increase Fe acquisition (Figures 1–3, A1). As could be expected, over-expression of *AtNAS* in NFP plants resulted in higher expression of *OsSAMS2*, *OsNAS1*, *OsNAS3*, and *OsDMAS1* as compared to the NTS plants, primarily under iron-deficient conditions (Figure A1).

*OsSAMS2* was expressed at higher levels during Fe deficiency (mainly in the early development stages) in both NFP and NTS plants, but at the milky stage of grain filling the NFP roots showed further 1.7-fold higher expression of *OsSAMS2* (Figure A1, Table 2) than NTS roots. At other stages and in other tissues tested, the NFP and NTS plants did not differ significantly for *OsSAMS2* expression. Among the *NAS* genes, *OsNAS3* was overall expressed at low levels compared to *OsNAS1* and *OsNAS2*, but its expression in NFP roots was significantly increased at mature stage under high iron condition and at the milky stage in the plants grown in iron-deficient conditions (Figure A1). In the mature stage of grain filling, *OsNAS1* was up-regulated in NFP roots (2.3-fold) as compared to NTS roots. *OsDMAS1* was generally up-regulated in iron-deficient conditions in both the genotypes, but at the milky and mature stages of grain filling, its expression was 2-fold and 1.8-fold higher in the NFP roots than NTS roots, respectively (Figure A1, Table 2). A significant increase of *OsDMAS1* expression could also be detected in NFP grains at the milky stage in iron-deficient conditions. These results suggest that the genes involved in NA and DMA synthesis are coordinately regulated in NFP plants, which contributed to increased iron uptake and facilitated translocation within these plants.

**Table 2:**
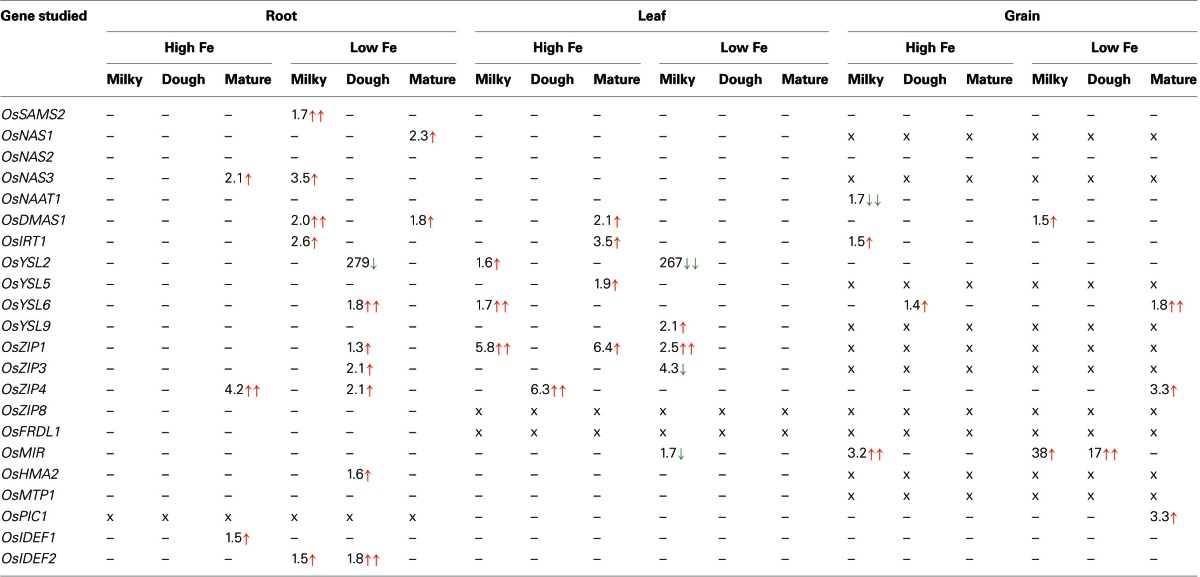
**Summary of expression differences obtained between NFP and NTS rice plants**.

For a particular gene studied, “–” represents no significant difference among the two genotypes, “x” represent no test made, and the values on the table represent fold changes observed in NFP plants as compared to NTS (statistically significant changes), where red upward arrows depict up-regulation while the downward green arrows depict down-regulation in the NFP plants, for a particular gene. Double arrows indicate significance at p ≤ *0.01* and single arrows indicate significance at p ≤ *0.05*. The milky, dough, and mature represent respective grain filling stages at which the plant material was collected.

### Other genes involved in iron uptake and iron translocation within the plant

The two iron-regulated transporters *OsIRT1* and *OsIRT2*, as well as several members of the YSL and zinc-regulated transporter IRT-like proteins (ZIP) family were studied, including *OsYSL2*, *OsYSL5*, *OsYSL6*, *OsYSL9*, *OsYSL13*, *OsYSL15* among YSLs and *ZIP1*, *ZIP3*, *ZIP4*, and *ZIP8* from the ZIP family (Figures 1–3).

**Figure A2:**
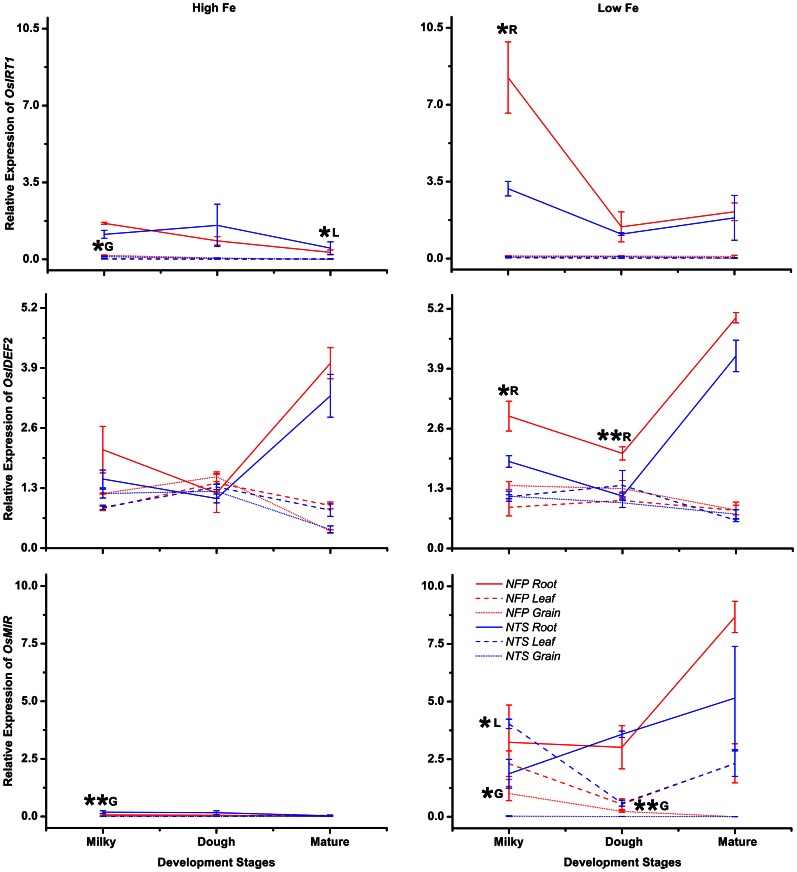
**Differences in expression levels of iron-regulated transporter *OsIRT1*, the transcription factor *OsIDEF2*, and the mitochondrial iron regulated gene *OsMIR*, in the roots, flag leaves, and grains of NFP vs. NTS plants are presented.** Values are mean ± standard error of mean, of the three biological replicates. Significant differences between NFP and NTS tissues (roots, flag leaves, grains) under a particular treatment (high Fe or low Fe) or at a particular development stage (milky, dough, or mature) are marked with single (^*^p ≤ 0.05) or double asterisks (^**^p ≤ 0.01), respectively. The letters R, L, and G represent root, leaf, and grain, respectively. Note the differences in scale at Y-axis for relative expression levels of tested genes.

Expression of *OsIRT1* and *OsIRT2* were mainly induced during iron deficiency and particularly in the roots, with *OsIRT1* expressed at higher levels than *OsIRT2* as was previously reported (Ishimaru et al., 2006). Further, *OsIRT1* was expressed 2.6-fold higher in NFP roots than NTS roots at the milky stage of grain filling (Figure A2, Table 2), while *OsIRT2* expression was not significantly different in NFP and NTS plants. *OsIRT1* was also significantly up-regulated in the grains (1.5-fold) and leaves (3.5-fold) of NFP plants growing under sufficient iron conditions, both at milky and mature stages of grain filling, respectively, but expression levels were generally low in these tissues.

**Figure A3:**
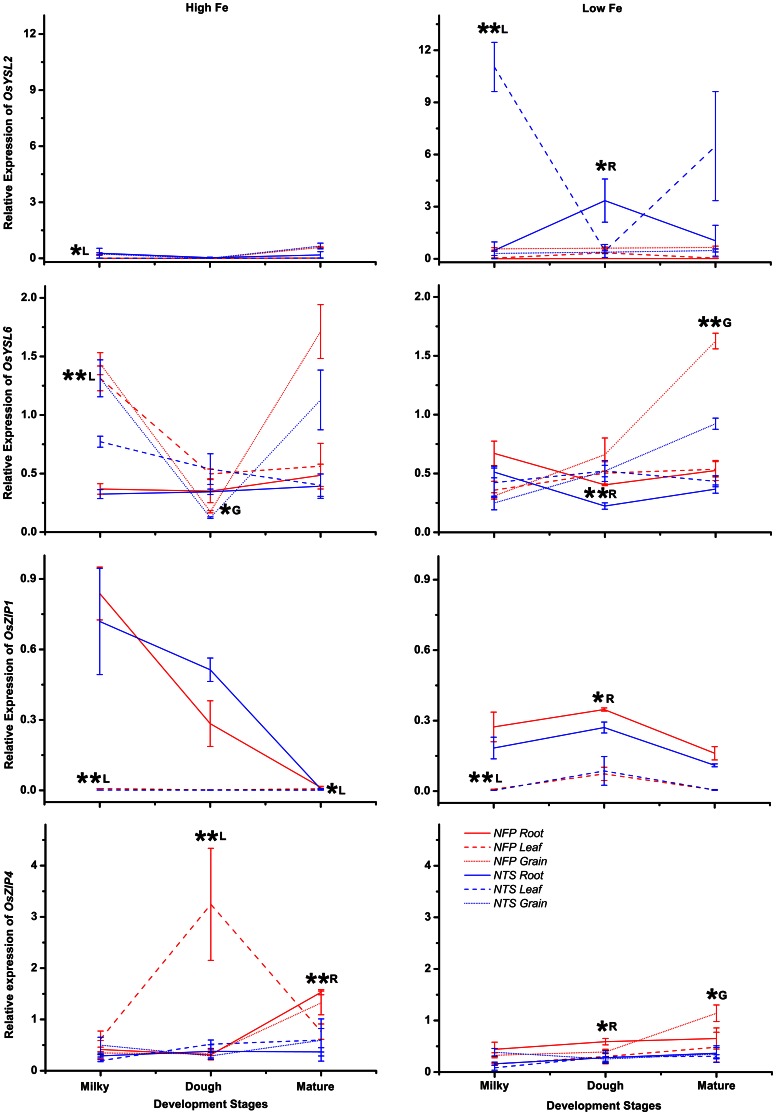
**Differences in expression levels of *OsYSL2* and *OsYSL6* (yellow stripe like transporters) as well as *OsZIP* and *OsZIP4* (zinc- and iron-regulated transporters), in the roots, flag leaves, and grains of NFP vs. NTS plants are presented.** Values are mean ± standard error of mean, of the three biological replicates. Significant differences between NFP and NTS tissues (roots, flag leaves, grains) under a particular treatment (high Fe or low Fe) or at a particular development stage (milky, dough, or mature) are marked with single (^*^p ≤ 0.05) or double asterisks (^**^p ≤ 0.01), respectively. The letters R, L, and G represent root, leaf, and grain, respectively. Note the differences in scale at Y-axis for relative expression levels of tested genes.

Among the *YSL* genes, significant transcript level differences were detected for *OsYSL2* and *OsYSL6*, while *OsYSL5*, *OsYSL9*, *OsYSL13*, and *OsYSL15* showed no or negligible expression differences between NFP and the NTS plants (Figures 1–3). *OsYSL2*, a transporter of the NA-Fe(II) complex, is induced during iron deficiency (Ishimaru et al., 2010), which is also the case in NTS plants where *OsYSL2* is significantly up-regulated in leaves at the milky stage and in the roots at the dough stage of grain filling (Figure A3, Table 2). However, the *OsYSL2* expression remained unchanged in NFP plants grown in low iron conditions. In contrast, a significant overexpression of *OsYSL6* was observed in NFP grains at maturity and a slightly increased expression in the roots at the dough stage of grain filling in iron-deficient conditions, as compared to NTS plants. Also, *OsYSL6* was up-regulated in NFP flag leaves at the milky stage of grain filling as compared to NTS leaves in iron-sufficient conditions. *OsYSL6* has been suggested as a Mn-NA transporter and also to play a role in detoxification of high Mn in roots and shoots (Sasaki et al., 2011). NFP and NTS plants contain similar Mn concentrations, except for some increase in polished and brown grains in iron-deficient conditions (Fe 20 μM; Wirth et al., 2009). It is possible that the induced expression of *OsYSL6* in NFP plants contributes to the small increase of Mn in NFP grains when iron availability is low.

*OsZIP4* was up-regulated in NFP leaves at dough stage of grain filling (6.3-fold) and in roots at grain maturity (4.2-fold) in iron-sufficient conditions. In iron-deficient conditions, NFP grains at maturity and NFP roots at the dough stage of grain filling had 3.3-fold and 2.1-fold higher expression of *OsZIP4* as compared to NTS plants, respectively (Figure A3, Table 2). *OsZIP1* was also up-regulated in low iron conditions in NFP roots at the dough stage (1.3-fold) and leaves at the milky stage of grain filling (2.5-fold). In addition, an approximate 6-fold higher expression was detected at the milky and mature stages of grain filling in the leaves of NFP plants grown with sufficient iron supply. However, it should be noted that *OsZIP1* is expressed only weakly in the leaves. *OsZIP3* and *OsZIP8* were expressed at even lower levels than *OsZIP1*, and their expression profiles were not significantly different between the genotypes, except for up-regulated *OsZIP3* expression in NFP roots at the dough stage of grain filling and down regulation in leaves at the milky stage of grain filling in low Fe conditions, as compared to NTS plants (Table 2). The upregulation of *OsZIP1* and *OsZIP4* under high iron conditions suggests that NFP plants signaled zinc deficiency when external iron concentration was high. Nevertheless, NFP plants perform better than the NTS plants in terms of zinc content in leaves and grains (Wirth et al., 2009) at both low and high iron conditions.

### Transcription factors and other inter- and intra-cellular transporters

The transcription factors encoded by *OsIDEF1* and *OsIDEF2* are known to be constitutively expressed and not affected by Fe deficiency (Kobayashi et al., 2007; Ogo et al., 2008). Similar expression patterns were obtained in our experiments, with an exception of *OsIDEF2*, which responded to iron deficiency in NFP plants. *OsIDEF2* expression was increased by 1.5- and 1.8-fold in NFP roots at milky and dough stages of grain filling as compared to NTS roots (Figure A2, Table 2). This up-regulation of *OsIDEF2* in NFP roots perhaps reinforced the Fe deficiency signal and thus led to up-regulation of genes involved in Fe translocation. *OsIDEF2* is known to be dominantly expressed in vascular bundles in the roots (Kobayashi et al., 2010b).

The mitochondrial iron-regulated (*OsMIR*) gene was mainly expressed upon iron deficiency. In comparison to the NTS plants, NFP grains had elevated expression of *OsMIR* at milky and dough stages of grain filling in iron-deficient conditions and at the milky stage of grain filling with sufficient iron availability. However, *OsMIR* was less induced in NFP leaves than the NTS leaves at the milky stage of grain filling (Figure A2, Table 2), but no significant expression differences were found at other stages. *OsHMA2*, *OsPIC1*, *OsMTP1*, *OsNRAMP4*, *OsFRDL1*, *OsFER1*, and *OsVIT2* did not show any significant differences between NFP and NTS plants (Figures 1–3). Only in iron-deficient conditions, NFP mature grains showed a higher expression of *OsPIC1* and NFP roots had higher *OsHMA2* expression at the dough stage of grain filling when compared to NTS plants (Table 2). *OsVIT2* was mostly expressed under sufficient iron conditions, with low expression detected under iron deficiency in roots and leaves. *OsVIT1* does not respond to Fe starvation while *OsVIT2* was found to be down-regulated in rice roots and shoots (Zhang et al., 2012).

**Figure 4:**
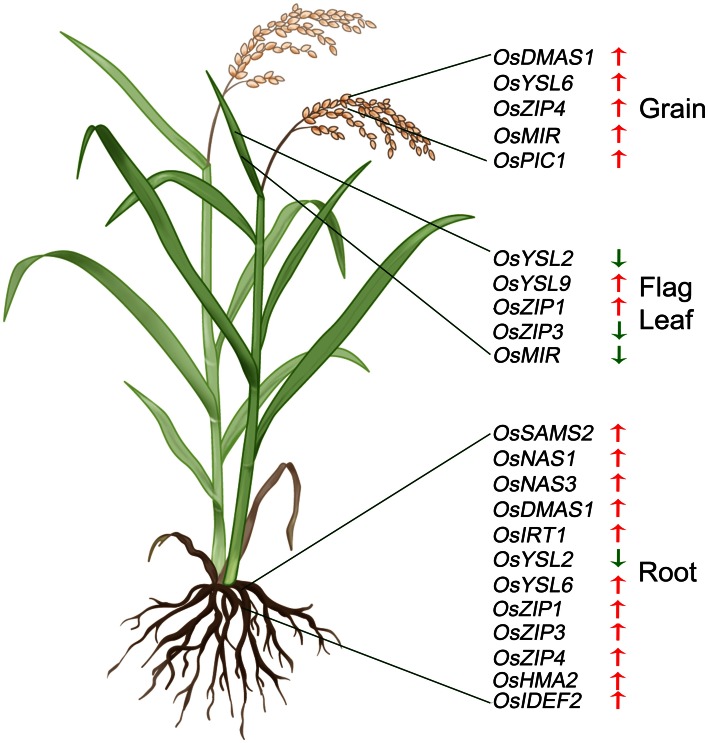
**Summary of significant expression changes observed in case of NFP plants vs. NTS plants, when subjected to low iron conditions.** Upward red arrows indicate up-regulated expression while downward green arrows indicate down-regulation of a particular gene, in the NFP plants as compared to the NTS plants. The genes are specifically marked according to the parts (roots, leaves, or grains) where the expression difference was observed.

The summary of genes that are differentially regulated in the NFP plants as compared to NTS plants, under Fe-deficient conditions is presented in Figure 4.

## Discussion

The combined overexpression of NAS and endosperm-specific expression of ferritin have a synergistic effect in increasing the iron content in the endosperm of NFP grains (Wirth et al., 2009). In the greenhouse, NFP plants show normal agronomic performance (e.g., plant height, tiller number, grain yield) and perform better under low Fe conditions than NTS plants (Wirth et al., 2009), suggesting that expression of *AtNAS1* and ferritin promoted iron increase in the endosperm without interfering with Fe homeostasis in NFP plants.

NA and DMA levels in plants are regulated by NAS and NAAT genes, and both NA and DMA are involved in Fe distribution in plants (Aoyama et al., 2009; Kakei et al., 2009). DMA/phytosiderophore synthesis involves successive reactions that are catalyzed by SAMS, NAS, NAAT, and DMAS (Bashir et al., 2010). *OsNAS1*, *OsNAS2*, and *OsNAS3* were proposed to perform different physiological functions in response to Fe deficiency (Inoue et al., 2003). Expression of *OsNAS1* and *OsNAS2*, both located on chromosome 3, is induced in rice roots exposed to Fe deficiency. Thus, a main role of these enzymes in NA synthesis appears to be the increased production of phytosiderophores in iron-deficient roots (Inoue et al., 2003). Expression of *OsNAS3*, which is located on chromosome 7, was found confined to pericycle cells close to protoxylem and companion cells, and was suggested to play rather limited role in phytosiderophore secretion from roots. Nevertheless, the OsNAS3 protein was shown to catalyze the trimerization of SAM to form NA (Inoue et al., 2003).

During low iron availability, expression of *OsSAMS2* and *OsNAS3* was significantly up-regulated at the milky stage of grain filling while *OsNAS1* had elevated expression levels at maturity in NFP roots when compared to NTS roots. This suggests that under iron deficiency, *AtNAS1* overexpression together with the enhanced expression of *OsNAS3* and *OsNAS1* resulted in increased NA synthesis at milky and mature stages of grain filling. NFP plants produce more NA under iron deficiency (Wirth et al., 2009). Since NA serves as the precursor for DMA, this increase in NA production most likely contributed to increased DMA content in NFP plants. Increased DMA production in NFP plants is further supported by the up-regulation of *OsDMAS1* in NFP roots, both at milky and mature stages of grain filling (Figure A1), as well as with increased DMA content in NFP leaves as compared to control plants (unpublished data). *OsNAS3* expression is also higher in NFP roots grown at high iron conditions, particularly at maturity. Together, these results suggest that the increased production of NA and DMA in NFP plants facilitated uptake of iron in roots and improved Fe translocation in the plants. In the roots of iron-deficient plants, *OsNAS1-3*, *OsNAAT1*, and *OsDMAS1* have similar expression patterns, with strong induction in pericycle cells adjacent to protoxylem (Inoue et al., 2003; Bashir et al., 2006; Inoue et al., 2008). Rice plants have increased DMA concentration in the xylem in iron-deficient conditions (Kakei et al., 2009). The specific contribution of *OsNAS3* to iron homeostasis has been previously reported (Lee et al., 2009b), where *OsNAS3* activation resulted in increased Fe and Zn concentration in the rice grains as well as increased tolerance to heavy metals. This increased Fe concentration is also well-correlated with increased NA and DMA content in plants that have increased *OsNAS3* expression (Lee et al., 2009b). Johnson et al. (2011) also showed a positive correlation between increased NA content and increased grain iron content in rice plants overexpressing either of *OsNAS1*, *OsNAS2*, and *OsNAS3*.

Furthermore, the demand for methionine is increased in iron-deficient plants in order to support the increased production of NA and then subsequently DMA. In our experiment, *OsSAMS2* was significantly up-regulated in roots of iron-deficient NFP and NTS plants, with a further expression increase in NFP roots at the milky stage of grain filling as compared to NTS plants (Figure A1). This increase of *OsSAMS2* expression would be expected to meet the demand for SAM, which is an immediate precursor for NA synthesis. Increased expression of genes participating in the methionine cycle in the roots of iron-deficient wheat, rice, and barley has been reported earlier (Ma et al., 1995; Negishi et al., 2002; Kobayashi et al., 2005).

In iron-deficient conditions, Fe needs to be effectively transported from roots to the shoots via the xylem, and then between cells. Significant advances have been made in identifying transporters involved in iron translocation. However, our understanding of the exact contribution of each one of these transporters in metal flux is rather limited. To date, the principal chelators known to bind iron include citrate (Rellan-Alvarez et al., 2010), NA and DMA (Takahashi et al., 2003; Aoyama et al., 2009; Kakei et al., 2009). The major role of DMA was initially considered to be in iron uptake from the rhizosphere, but several lines of evidence support a chelating role of DMA in both xylem and phloem (Aoyama et al., 2009; Kakei et al., 2009). Based on the expression of *OsDMAS1* it has been proposed that DMA is synthesized in the phloem companion cells (Inoue et al., 2008). High concentrations of DMA have been detected in phloem sap in independent studies advocating the involvement of DMA in long distance iron transport (Nishiyama et al., 2012). Several transporters belonging to YSL family could be transporting these chelator-bound iron complexes to other parts of the plant. OsYSL15 and OsYSL18 transport Fe(III)-DMA complexes and are involved in internal translocation of iron (Aoyama et al., 2009; Inoue et al., 2009), while OsYSL2 transports Fe(II)-NA and Mn(II)-NA complexes, but not Fe(III)-DMA (Koike et al., 2004; Ishimaru et al., 2010).

*OsYSL2* is induced by iron-deficiency and may be actively involved in long distance phloem transport of Fe(II)-NA complexes in the plant and into the grains (Koike et al., 2004; Ishimaru et al., 2010). Rice plants with reduced *OsYSL2* function (RNAi-OsYSL2) have reduced Fe and Mn concentrations in the grains (Ishimaru et al., 2010). Consistent with previous reports (Ishimaru et al., 2010), NTS plants showed induced expression of *OsYSL2* upon iron deficiency, but the gene was not up-regulated in NFP roots and leaves under the same condition (Figure A3). In the grains, however, the expression of *OsYSL2* increased in NFP plants as well but was not significantly different than in NTS grains. A plausible explanation for this result could be that expression of *OsYSL2* is regulated by the endogenous iron status of the plants. It has also been suggested that OsIDEF2 directly regulates expression of *OsYSL2* (Ogo et al., 2008). However, the up-regulation of *OsIDEF2* in iron-deficient NFP roots (Figure A2) did not lead to increased *OsYSL2* expression. It is possible that OsIDEF2 also senses cellular iron status in order to induce the iron-deficiency responsive genes, as was suggested for OsIDEF1 which binds directly to divalent metals for sensing cellular metal ion balance (Kobayashi et al., 2012). Importantly, the NFP plants had higher iron content in the grains as compared to NTS plants. It is also possible that NFP plants deployed alternate modes of iron transport to grains than the transfer by OsYSL2 and that the function of OsYSL2 is complemented by another transporter. Our results also reflect effective crosstalk between molecular components involved in Fe homeostasis in different growth conditions and during development to meet the needs for Fe in the plant.

*OsMIR*, a recently evolved rice-specific mitochondrial gene, is strongly induced under iron deficiency (Ishimaru et al., 2009). NFP leaves had lower expression levels of *OsMIR* as compared to NTS leaves under low iron conditions, but the gene was up-regulated in NFP grains (Figure A2). Since mitochondrial Fe regulation is poorly understood, it is difficult to predict how and to what extent these expression differences contributed to higher grain iron content in the NFP plants. In addition, NFP leaves also had increased expression of *OsZIP1* and *OsZIP4* under high iron conditions, indicating that NFP plants might signal zinc deficiency when external iron concentration is high. Although *OsZIP4* is regulated by zinc (Ishimaru et al., 2005), there exists a strong crosstalk between Zn and Fe homeostasis in plants. Fe concentrations doubled in Zn-deficient roots (Ishimaru et al., 2005) and plants overexpressing *OsZIP4* had significantly increased Fe in the shoots and roots, in addition to the Zn increases (Ishimaru et al., 2007). This demonstrates the coordination of Fe and Zn homeostasis in plants and also that the expression of *OsZIP1* and *OsZIP4* are affected by the external supply of iron to the plants (as in the case of both NFP and NTS plants) as well as by endogenous Fe nutritional status of plants (further increases in NFP plants). Nevertheless, NFP plants had a similar zinc content in the leaves as compared to the controls over a range of tested external iron concentrations and outperformed NTS plants in terms of zinc content in the grains (Wirth et al., 2009). Therefore, Zn homeostasis is also unaffected in NFP plants.

Together, the increased production of NA and DMA in NFP plants facilitated iron uptake from the rhizosphere as well as effective internal translocation. Expression of several transporter genes appear to be adjusted in the NFP plants in order to utilize overproduced NA and DMA, and the expanded sink for iron storage in the grains via ferritin. However, these adjustments did not interfere with Fe homeostasis in the NFP plants. Further investigations focused on iron speciation in the grain, i.e., Fe(II) or Fe(III), and the relative abundance of two forms together with the information on molecules chelated to these forms, will be required for elucidating the exact mechanisms of iron translocation to the grains in NFP plants.

## Materials and methods

### Plant material

The NFP plants and their non-transgenic siblings (NTS) were grown under greenhouse conditions, in the hydroponics system. The NFP plants are the *Oryza sativa* ssp. japonica cv. Taipei 309 transformed with *Arabidopsis Nicotianamine Synthase* gene, *Phaseolus vulgaris Ferritin*, and *A. fumigatus Phytase* gene (NFP plants; Wirth et al., 2009). Solutions for the hydroponic system were prepared according to the protocol modified from Kobayashi et al. (2005), using 0.70 mM K_2_SO_4_, 0.10 mM KCl, 0.10 mM KH_2_PO_4_, 2.0 mM Ca(NO_3_)_2_, 0.50 mM MgSO_4_, 10 μM H_3_BO_3_, 0.50 μM MnSO_4_, 0.20 μM CuSO_4_, 0.01 μM (NH4)_6_Mo_7_O_24_, and 0.5 μM ZnSO_4_, with different iron concentrations added as Fe(III)-EDTA according to the treatment (high iron condition: 200 μM iron; iron-deficient condition: 10 μM iron). Samples were collected at three different grain filling stages: milky stage, dough stage, and mature stage. At the milky stage grains are starting to fill with a white, milky liquid that can be squeezed by pressing the grain between fingers, while in dough stage the milky portion of grain turns into a soft dough and at maturity, the grain is fully developed and hard (parameters as defined in the Rice Knowledge Bank, IRRI, Philippines). At each developmental stage, roots, flag leaves and grains were collected, with at least three biological replicates.

### Total RNA extraction and cDNA synthesis

Total RNA was extracted from the root, flag leaf, and grain samples using Trizol® reagent (Invitrogen, USA) and was treated with DNase I (Thermo Fisher Scientific Inc., USA). RevertAid™ first strand cDNA synthesis kit (Thermo Fisher Scientific Inc., USA) was used for cDNA synthesis. All steps were carried out following the manufacturers' instructions.

### Real-time quantitative PCR

Real-time quantitative PCRs (qRT-PCR) were carried out using Taqman hydrolysis probes (Roche, Switzerland) on 7500 FAST Real Time PCR system (Applied Biosystems, Inc., USA). Total reaction volume of 25 μl was used, comprising of 12.5 μl mastermix (Applied Biosystems Inc., USA), 1 μl cDNA, 2.25 μl forward primer and 2.25 μl reverse primer, 0.25 μl probe (Roche Ltd., Switzerland) and 6.75 μl H_2_O.

Primers were designed using Roche primer design website (https://www.roche-applied-science.com/sis/rtpcr/upl/index.jsp?id=UP030000). Probe number and primer sequences are presented in the Appendix (Table A1). The Ct value was obtained from 7500 Fast System Software (Applied Biosystems, Inc., USA). The primer efficiency was calculated using LinReg PCR (Tuomi et al., 2010). qRT-PCR data normalization was done as described by Schefe et al. (2006). The obtained data were further analyzed by ANOVA and significant differences between the tested plant materials are presented.

### Conflict of interest statement

The authors declare that the research was conducted in the absence of any commercial or financial relationships that could be construed as a potential conflict of interest.

## Appendix

**Table A1:** **Primers and probes list for real-time quantitative PCR**.

**Nr.**	**Gene**	**Accession ID**	**Forward primer**	**Reverse primer**	**TaqMan probe**
1	*OsSAMS2*	U82833	ccgaataaaggcgagaagc	gactcggaggtgaagaggaa	70
2	*OsNAS1*	AB021746	cggttgagaaggcagaagagt	cgatcgtccggctgttag	17
3	*OsNAS2*	AB023818	cacctcgaggcgcactac	tccagcttgctcaggttga	149
4	*OsNAS3*	AB023819	gaggaggaggtgatcgagaa	atcaccagctccgtgaaca	70
5	*OsNAAT1*	AB206814	ttgccaacttgcgaaagaa	aatgcgaacccaattcttca	61
6	*OsDMAS1*	AB269906	aaaagctcgacaccctgct	tccctcagcttcctctgct	39
7	*OsIRT1*	AB070226	gacactggtgcccattctg	gaggatggggatggagga	63
8	*OsIRT2*	AB126086	tcaggaatcgcgtcattgt	agcccgatcaccactgag	105
9	*OsYSL2*	AB164646	tggagcttcttccagtggtt	gaggctgaaatcaaaatagaacg	22
10	*OsYSL5*	AB190915	agcttgcatggaaaaacagaa	aaagaagctccagccaaaact	4
11	*OsYSL6*	AB190916	gaacaccgtcatccagacct	gttttctgatccattgcaagc	141
12	*OsYSL9*	AB190919	tgcgtggatgactggattc	tcccacttgggtaagttaatttgt	152
13	*OsYSL13*	AK067235	atccagacctgcgtcgtc	catggacaatatgtagttaccaaagc	161
14	*OsYSL15*	AB190923	tcgcctgctacaccatagc	cagctcgtacgtcctcttgtt	107
15	*OsZIP1*	AY302058	gtgatgagccgcaaggag	cctcgaacgacgatgtcc	159
16	*OsZIP3*	AY323915	tgcatctgtgaggccatcta	tgacggttgcccttacctta	31
17	*OsZIP4*	AB126089	gtcaatcaggccactcgtc	gctttcgccttaaaatttgc	31
18	*OsZIP8*	AK070864	atcagttcttcgagggcatc	gcgtcgtagacggaggag	143
19	*OsFRDL1*	NM_001055921	gactccacttcgatccacaaa	ctcgctccgcttatcacg	146
20	*OsNRAMP4*	AK102180	gccattggcttcctagatcc	aagatgacccacagaagctca	78
21	*OsMIR*	AK103636	acatttatcctactcgttgctcct	tgcctaagtgctgagtcacg	43
22	*OsHMA2*	HQ646362	gtcaacatactcatgctgattgc	cccagcctcagaatagtcctt	67
23	*OsMTP1*	AK100735	tcaatctccatcaccatcca	tcactcttgagcaatggttcc	157
24	*OsVIT2*	Os09g0396900	ggcctcggagggtatctg	acagtatgtccgcgatctcc	15
25	*OsPIC1*	XM_464386	accgagcaggacatcgag	ggaaacaactgtggacacca	93
26	*OsFER1*	AF519570	aggggatgccttgtatgct	cggtcagctgtggatcatt	148
27	*OsIDEF1*	AK107456	gtcttcaggctggggatgt	gggatttgttgtctgctgatg	91
28	*OsIDEF2*	AK099540	cagatgttgaactgtataaatttgctc	ttcaagatctctgctctggagac	15
